# Association of Serum Zinc Level with severity of chronic kidney disease in diabetic patients: a cross-sectional study

**DOI:** 10.1186/s12882-022-03040-x

**Published:** 2022-12-21

**Authors:** Mitsunobu Kubota, Shizuka Matsuda, Mimu Matsuda, Kazuki Yamamoto, Yoko Yoshii

**Affiliations:** Department of Endocrinology and Diabetes, National Hospital Organization Kure Medical Center and Chugoku Cancer Center, 3-1 Aoyamacho, Kure, Hiroshima, 737-0023 Japan

**Keywords:** Serum zinc concentration, Diabetic nephropathy, Diabetic kidney disease, Chronic kidney disease, Estimated glomerular filtration rate, Albuminuria

## Abstract

**Background:**

In recent years, it has been reported that diabetic patients tend to have a lower zinc intake due to unbalanced diet accompanying changes in lifestyle habits. We investigated serum zinc concentration in diabetic patients according to the stage of nephropathy.

**Methods:**

We enrolled 227 diabetic patients (119 men, 108 women, average age 65.7 ± 14.7 [mean ± standard deviation]) who were hospitalized for diabetes treatment due to poor blood glucose control. We investigated the relationship between fasting serum zinc concentration and estimated glomerular filtration rate (eGFR) and albuminuria (urinary albumin-to-creatinine ratio, UACR), as well as serum zinc concentration by stage of diabetic kidney disease and chronic kidney disease.

**Results:**

The mean HbA1c value was 10.5 ± 2.1%. Serum zinc concentration was 75.5 ± 16.0 μg/dL in males and 75.7 ± 12.2 μg/dL in females, showing no gender difference and no significant relationship with diabetes type. The serum zinc concentration was negatively correlated with age (r = − 0.309, *P* < 0.001) and positively correlated with eGFR (r = 0.144, *P* = 0.030). A tendency was observed of serum zinc concentration to decrease after overt nephropathy, with values of 76.4 ± 14.1 μg/dL in pre-nephropathy (stage 1, *n* = 131), 78.5 ± 13.2 μg/dL in incipient nephropathy (stage 2, *n* = 65), 66.4 ± 14.3 μg/dL in overt nephropathy (stage 3, *n* = 25), and 65.7 ± 11.9 μg/dL in kidney failure (stage 4, *n* = 6). Serum zinc showed a negative trend with estimated GFR (*P* = 0.004) and significant reduction in albuminuria, with stage A3 (*n* = 29, 65.7 ± 13.9 μg/dL) having lower levels than A1 (*n* = 131, 76.4 ± 14.1 μg/dL, *P* = 0.001) and A2 (*n* = 67, 78.4 ± 13.1 μg/dL, *P* < 0.001).

**Conclusions:**

In diabetic patients, serum zinc concentration tended to decrease as age increased and also as renal function deteriorated. This study suggests that consideration of zinc deficiency is necessary in patients with overt albuminuria.

**Supplementary Information:**

The online version contains supplementary material available at 10.1186/s12882-022-03040-x.

## Background

Type 2 diabetes is on the rise worldwide [1]. In recent years, it has been reported that diabetes patients tend to have lower zinc intake due to an unbalanced diet resulting from changes in lifestyle habits and an increased rate of eating disorders, especially among women [2]. Zinc is abundant in oysters, beef, liver, seafood, cereals, beans, nuts, and cheese [2], and is important as a cofactor for many enzymes, including the antioxidant enzymes catalase, peroxidase, and superoxide dismutase [3]. Diabetic patients sometimes consume a lot of processed foods [4], which may lose zinc content in the course of processing. Furthermore, polyphosphate and phytic acid, two additives used in processed foods, are known to chelate zinc and inhibit its absorption from the intestinal tract [5]. Although few reports have indicated direct association of zinc deficiency with diabetes, a cohort study in the United States has shown that women with low zinc intake have increased risk of developing diabetes [6]. Moreover, zinc deficiency exacerbates insulin resistance in non-insulin-dependent diabetics [7], and serum zinc levels are both inversely correlated with HbA1c levels in diabetic patients [8] and associated with diabetic peripheral neuropathy [9]. It has been reported that even when zinc intake is about 10 mg/day, diabetic patients are likely to become zinc deficient [10]. In these patients, mechanisms of zinc deficiency include breakdown of fat and muscle due to diabetes progression (with consequent excretion of the zinc contained in those tissues), impaired zinc absorption in the gastrointestinal tract [11], and renal damage due to diabetic nephropathy, which increases urinary zinc excretion; additionally, if proteinuria increases in diabetic kidney disease (DKD), zinc loss also increases [12, 13]. Zinc deficiency can cause various disorders in diabetic patients, such as anemia, delayed wound healing, decreased reproductive function, decreased sense of taste, dermatitis, stomatitis, and hair loss [14]; accordingly, it is considered necessary to pay attention to serum zinc concentration in diabetic patients.

DKD can involve the kidneys in different forms. The primary form is diabetic nephropathy, a microvascular complication of diabetes that progresses to microalbuminuria, overt albuminuria, and decreased renal function, which is associated with increased mortality [15]. Other forms of DKD can induce decreased eGFR without development of albuminuria [16]. Chronic kidney disease (CKD) is a prevalent cause of renal involvement, affecting up to 30–40% of patients with type 2 diabetes mellitus in Europe [17] and 7–20% in Asia [18]. In Japan, the prevalence of DKD in patients with type 2 diabetes is reported to be 42% [19]. Fukushima et al. reported that hypozincemia may be involved in renal anemia, which is difficult to improve even with external erythropoietin agents, because the serum zinc concentration is low in dialysis patients [20]. Zinc may also be associated with diabetic kidney disease [21]; in fact, a positive correlation was observed between the estimated glomerular filtration rate (eGFR) and serum zinc concentration in patients with chronic kidney disease (CKD) [22], and there is a possibility that diabetic renal failure is accompanied by decreased serum zinc level [23]. However, as far as we know, no reports have examined the relationship between serum zinc concentration and detailed DKD stage in diabetic patients. Therefore, we investigated serum zinc concentration in diabetic patients according to diabetic nephropathy and CKD stage.

## Methods

### Study group

We enrolled 227 diabetic patients (119 men, 108 women, average age 65.7 ± 14.7 [mean ± standard deviation]) who were hospitalized in our department during the period from April 2018 to April 2020 for diabetes treatment due to poor blood glucose control. A retrospective analysis was performed on 17 patients with type 1 diabetes, 204 with type 2 diabetes, and 6 with diabetes of other etiologies. For each patient, we recorded body weight, blood pressure, glucose metabolism index, lipid metabolism index, eGFR, calculated by the Chronic Kidney Disease Epidemiology Collaboration (CKD-EPI) formula [24], albuminuria related to DKD (urinary albumin-to-creatinine ratio, UACR, with albumin measured in milligrams and creatinine in grams), and serum zinc concentration. Each participant was interviewed and provided informed consent. This study was approved by the Ethics Committee of the National Hospital Organization of Kure Medical Center (file number 30–41).

### Biochemical analyses

After overnight fasting, each participant underwent a physical examination and venous blood collection early in the morning. Body measurements were taken in the standing position. Body mass index (BMI) was calculated as weight (kg) / height (m)^2^. Body fat, body fat percentage, muscle mass, and skeletal muscle mass were measured by the bioelectrical impedance method using multifrequency BIA (MF-BIA; InBody 770, Cerritos, CA, USA). The height-adjusted skeletal muscle mass index (SMI: skeletal muscle mass index, [kg]/square of height [m^2^]) was calculated based on the obtained body weight for statistical analyses [25]. Collected blood and urine samples were centrifuged and measured by each method. Plasma glucose levels were measured by the glucose oxidase method and HbA1c levels by high performance liquid chromatography (HPLC) on a HLC723-G9 (Tosoh, Tokyo, Japan; range of measurement, 1.9–18.6%; coefficient of variation [CV], 0.58%). Serum and urine creatinine levels were measured by the oxidase method (Sekisui Medical, Tokyo, Japan; limit of detection [LOD], 0.029 mg/dL; limit of quantitation [LOQ-CV10%], 0.205 mg/dL; range of measurement, 0.05–100 mg/dL; CV, 0.4%), and values in mL/min/1.73 m^2^ calculated as follows: 194 x Serum creatinine (− 1.094) x Age (− 0.287) × 0.739 (if female) [24]. Urine albumin was quantified using the immunoturbidimetry method (Nittobo Medical, Tokyo, Japan; LOD, 0.183 μg/mL; LOQ-CV10%, 0.697 μg/mL; range of measurement, 1.0–500 μg/mL; CV, 0.86%). Serum total cholesterol (TC) and triglyceride (TG) levels were assessed with an enzymatic method (Sekisui Medical, Tokyo, Japan; TC: LOD, 0.03 mg/dL; LOQ-CV10%, 0.17 md/dL; range of measurement, 5–1000 mg/dL; CV, 0.46%; TG: LOD, 0.20 mg/dL; LOQ-CV10%, 0.40 md/dL; range of measurement, 3–2000 mg/dL; CV, 0.68%). High-density lipoprotein cholesterol (HDL-C) was measured directly by homogeneous assay (Sekisui Medical, Tokyo, Japan; LOD, 0.07 mg/dL; LOQ-CV10%, 0.13 md/dL; range of measurement, 2–150 mg/dL; CV, 0.59%). Low-density-lipoprotein cholesterol (LDL-C) was determined using the Friedewald equation [26]. Serum zinc concentration was measured using the ACCURAS AUTO Zn reagent kit (Shino-Test Corporation, Japan; LOD, 1.87 μg/dL; LOQ-CV10%, 5.312 μg/dL; range of measurement, 4.0–500 μg/dL; CV, 1.08%), which can be employed with all auto-analyzers widely used in hospital laboratories and does not need any serum pretreatment [27]. Serum zinc levels were categorized according to the criteria of the Japanese Society of Clinical Nutrition [28]: normal, ≥80 μg/dL; subclinical zinc deficiency, ≥60 μg/dL and < 80 μg/dL; and zinc deficient, < 60 μg/dL. DKD was categorized into stages according to the 2014 classification of the Joint Committee on Diabetic Nephropathy [29]: stage 1 (pre-nephropathy), normoalbuminuria (A1) < 30 mg/gCr and eGFR ≥30 mL/min/1.73m^2^; stage 2 (incipient nephropathy), microalbuminuria (A2) 30–299 mg/gCre and eGFR ≥30 mL/min/1.73m^2^; stage 3 (overt nephropathy), overt albuminuria (A3) ≥300 mg/gCr and eGFR ≥30 mL/min/1.73m^2^; stage 4 (kidney failure), eGFR < 30 mL/min/1.73m^2^ and not on dialysis; and stage 5 (dialysis therapy), end-stage renal failure regardless of albuminuria status.

### Statistical analysis

Data are expressed as mean ± S.D. or median (25th–75th percentiles) depending on the data distribution. Due to having skewed distributions, TG, eGFR, and UACR values were logarithmically transformed before analysis. Differences in continuous variables between subcategories were first tested for significance using analysis of covariance, and if they were found to be significant, the Tukey-Kramer method was used to assess difference between categories. Categorized variables were analyzed using the *χ*^2^ test. Spearman’s correlation coefficient (r) and *P*-values are given for univariate correlation of serum Zn with metabolic variables. *P-*values < 0.05 were considered statistically significant. All analyses were performed using the software package SPSS version 27 (IBM Co. Ltd., Armonk, NY, USA).

## Results

**Fig. 1:**
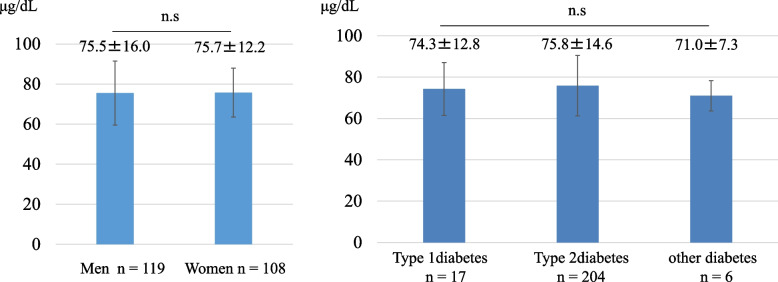
Serum zinc concentration in diabetic patients by gender and diabetes typeSerum zinc concentrations were 75.5 ± 16.0 μg/dL in males (*n* = 119) and 75.7 ± 12.2 μg/dL in females (*n* = 108), with no statistically significant difference. Values by diabetes type were: 74.3 ± 12.8 μg/dL for type 1 diabetes (*n* = 17), 75.8 ± 14.6 μg/dL for type 2 diabetes (*n* = 207), and 71.0 ± 7.3 μg/dL for diabetes due to other etiologies (*n* = 6). There was no statistically significant difference by diabetes type. Significance was determined by analysis of covariance. Columns and error bars indicate mean ± standard deviation (S.D.)

The study subjects consisted of 17 with type 1 diabetes, 204 with type 2 diabetes, and 6 with other etiologies. The average HbA1c was 10.5 ± 2.1%, indicating poor glycemic control. The average serum zinc level was 75.6 ± 14.3 μg/dL, and according to the criteria of the Japanese Society of Clinical Nutrition [28], 96 were normal, 103 were subclinical zinc deficient, and 28 were zinc deficient. Overall, 57.7% of patients had zinc deficiency or subclinical zinc deficiency (Table 1). Serum zinc concentrations were 75.5 ± 16.0 μg/dL in males and 75.7 ± 12.2 μg/dL in females, and exhibited no gender difference and no significant relationship with diabetes type (Fig. 1). Serum zinc concentration was negatively correlated with age (r = − 0.354, *P* < 0.001) and positively correlated with body weight (r = 0.170, *P* = 0.007), SMI (r = 0.199, *P* = 0.003). It also showed both a positive correlation with eGFR(r = 0.144, *P* = 0.030) and a negative correlation with the GFR classification of CKD (r = − 0.137, *P* = 0.037). There was no significant association of serum zinc with HbA1c, an index of blood glucose control (Table 2).

**Table 1:** Baseline characteristics of participants

N (Men/ Women)	227 (119/ 108)
Diabetes subtype (Type 1/ Type 2/ other)	227 (17/ 204/ 6)
Age, *years*	65.7 ± 15.5
Height, *cm*	159.2 ± 9.4
Body weight, *kg*	64.5 ± 15.5
BMI, *kg/m*^*2*^	25.3 ± 4.9
Fasting glucose, *mg/dl*	176.9 ± 63.7
HbA1c, *%*	10.5 ± 2.1
TC, *mg/dl*	185.5 ± 45.1
LDL-C, *mg/dl*	106.9 ± 35.2
TG, *mg/dl*	139.0 (91.0–176.0)
HDL-C, *mg/dl*	47.3 ± 14.8
Non HDL-C, *mg/dl*	138.6 ± 43.3
Zn, *μg/dL*	75.6 ± 14.3
Estimated GFR, *ml/min/1.73m*^*2*^	75.7 (55.4–88.9)
Urinary albumin-to-creatinine ratio, *mg Alb /g Cre*	20.0 (7.0–67.0)
Diabetic nephropathy stage (1/ 2/ 3/ 4/ 5)	131/ 65/ 25/ 6/ 0
CKD stage	
GFR (G1/ G2/ G3a/ G3b/ G4/ G5)	54/ 105/ 39/ 23/ 5/ 1
incidence of albuminuria (A1/ A2/ A3)	131/ 67/ 29
Diabetic retinopathy stage	
(NDR/ SDR/ PPDR/ PDR)	128/ 37/ 9/ 11
Smoking (None/ Past/ Current)	121/ 60/ 46
Serum zinc classification	
(Normal/ Subclinical deficiency/ Deficiency)	96/ 103/ 28

*BMI* body mass index, *HbA1c* hemoglobin *A1c*, *TC* total cholesterol, *LDL* low-density lipoprotein, *HDL* high-density lipoprotein, *TG* triglyceride, *GFR* glomerular filtration rate, *CKD* chronic kidney disease, *NDR* no diabetic retinopathy, *SDR* simple diabetic retinopathy, *PPDR* pre-proliferative diabetic retinopathy, *PDR* proliferative diabetic retinopathy; serum zinc classification: normal, ≥80 μg/dL; subclinical deficiency, ≥60 and <80 μg/dL, deficiency, <60 μg/dL

Data are presented as number, mean ± S.D., or median (25th–75th percentiles)

**Table 2:** Relationship between serum zinc concentration and metabolic parameters in all subjects

Serum zinc concentration		
	*r*	*P*
Age, *years*	−0.354	< 0.001
Body weight, *kg*	0.170	0.007
BMI, *kg/m*^*2*^	0.077	0.247
SMI	0.199	0.003
Fasting glucose, *mg/dl*	0.036	0.591
HbA1c, *%*	−0.070	0.301
TC，*mg/dl*	0.245	0.001
LDL-C, *mg/dl*	0.134	0.044
TG，*mg/dl*	0.170	0.010
HDL-C, *mg/dl*	0.083	0.241
Non HDL-C, *mg/dl*	0.205	0.004
Estimated GFR, *ml/min/1.73m*^*2*^	0.144	0.030
Urinary albumin-to-creatinine ratio, *mg Alb /g Cre*	−0.078	0.241
Diabetic nephropathy stage (1/ 2/ 3/ 4/ 5)	−0.109	0.100
CKD stage		
GFR (G1/ G2/ G3a/ G3b/ G4/ G5)	−0.137	0.039
Albuminuria (A1/ A2/ A3)	−0.109	0.102

Univariate correlations of serum zinc concentration with measured variables in all subjects. Spearman’s rank correlation coefficients (r) and P-values are presented. BMI body mass index, HbA1c hemoglobin A1c, TC total cholesterol, LDL low-density lipoprotein, HDL high-density lipoprotein, TG triglyceride, GFR glomerular filtration rate, CKD chronic kidney disease, SMI, skeletal muscle index

Data are presented as number, mean ± S.D., or median (25th–75th percentiles)

**Fig. 2:**
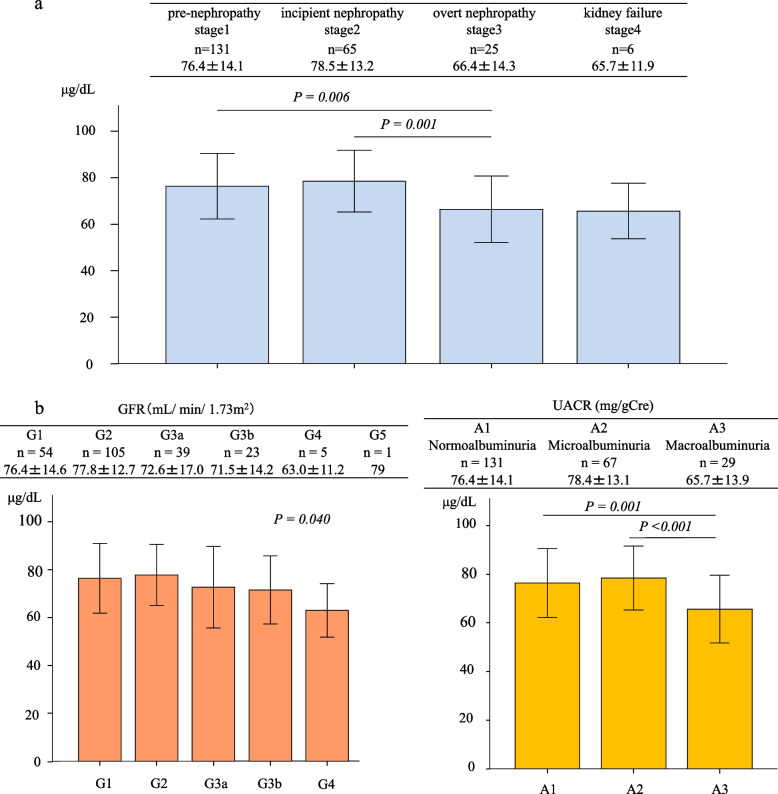
**a** Serum zinc concentration by diabetic nephropathy stage. Diabetic patients were classified by stages according to the 2014 classification of the Joint Committee on Diabetic Nephropathy [29] and serum zinc concentrations compared. Stages and concentrations were as follows: pre-nephropathy (stage 1, *n* = 131), 76.4 ± 14.1 μg/dL; incipient nephropathy (stage 2, *n* = 65), 78.5 ± 13.2 μg/dL; overt nephropathy (stage 3, n = 25), 66.4 ± 14.3 μg/dL; kidney failure (stage 4, *n* = 6), 65.7 ± 11.9 μg/dL. Serum zinc levels were significantly lower in overt nephropathy than in pre-nephropathy (*P* = 0.006) and incipient nephropathy (*P* = 0.001). Significance was determined by analysis of covariance and the Tukey-Kramer method was used to assess difference between categories. Columns and error bars indicate mean ± standard deviation (S.D.). **b** Serum zinc concentration and CKD severityDiabetic patients were classified by CKD severity and serum zinc concentrations compared. A weak negative trend was observed in relation to the GFR segment (*P* = 0.004) and a significant difference in relation to albuminuria category, with stage A3 (*n* = 29, 65.7 ± 13.9 μg/dL) featuring lower serum zinc than either A1 (*n* = 131, 76.4 ± 14.1 μg/dL, *P* = 0.001) or A2 (*n* = 67, 78.4 ± 13.1 μg/dL, *P* < 0.001). Significance was determined by analysis of covariance, and if significant, the Tukey-Kramer method was used to assess difference between categories. Columns and error bars indicate mean ± standard deviation (S.D.)

Categorized by stage of diabetic nephropathy [29], 131 participants were in stage 1, 65 in stage 2, 25 in stage 3, and 6 in stage 4 (Table 1). The corresponding serum zinc concentrations for each group were 76.4 ± 14.1 μg/dL, 78.5 ± 13.2 μg/dL, 66.4 ± 14.3 μg/dL, and 65.7 ± 11.9 μg/dL, with a tendency of serum zinc to decrease after overt nephropathy (stage 3) compared to pre-nephropathy (stage 1) and incipient nephropathy (stage 2) (Fig. 2a). Similarly examining serum zinc concentration by CKD severity classification [30] revealed a weak negative trend in the GFR segment (*P* = 0.004) and significant difference in the albuminuria segment, with lower serum zinc in stage A3 (macroalbuminuria, *n* = 29, 65.7 ± 13.9 μg/dL) than in either A1 (normoalbuminuria, *n* = 131, 76.4 ± 14.1 μg/dL, *P* = 0.001) or A2 (microalbuminuria, *n* = 67, 78.4 ± 13.1 μg/ dL, *P* < 0.001) (Fig. 2b).

Furthermore, since serum zinc was found to be correlated with SMI in diabetic patients (Table 2), we investigated the relationship between SMI and age and serum zinc level. SMI tended to decrease with age in both men and women (Supplemental Fig. 1). A tendency was also observed for SMI to be low in zinc-deficient subjects, especially males, and both male and female zinc-deficient participants in this Japanese population exhibited SMI values meeting the criteria for sarcopenia set by the Asian Sarcopenia Working Group [31] (less than 7.0 kg/m^2^ for men and 5.7 kg/m^2^ for women; Supplemental Fig. 2).

## Discussion

In diabetic patients, serum zinc concentration tended to decrease as age increased and as renal function deteriorated. The results particularly suggest that consideration of zinc deficiency is necessary in patients having nephropathy with overt albuminuria.

In diabetic patients, diet restriction, unbalanced diet, and insufficient protein intake may reduce serum zinc concentration. Especially for patients having nephropathy with overt albuminuria, nutritional guidance recommends avoiding excessive intake of foods rich in animal protein [32]. However, animal-based foods show high bioavailability, and should be a good source of zinc [2]. Therefore, a protein-restricted diet in patients with DKD may result in reduced intake of zinc-rich animal protein and reduction of pooled zinc in the body [33], even if the zinc absorption rate of the body increases to compensate for the decreased intake [34]. In other words, nutritional guidance for DKD should also consider the intake of trace elements such as zinc. In addition, this study found serum zinc concentration to be positively correlated with body weight, skeletal muscle index, and serum cholesterol level in diabetic patients (Table 2). This suggests that serum zinc reflects dietary intake and nutritional status. It is well known that zinc deficiency causes dysgeusia [35], producing a vicious circle in which dysgeusia reduces appetite and causes further zinc deficiency. Zinc supplementation in patients with renal failure and hemodialysis has been reported to increase serum zinc levels, reduce the rate of protein catabolism, and improve malnutrition [36]. Accordingly, it is important to screen for hypozincemia.

Under hyperglycemia, production of free radicals such as superoxide is enhanced, resulting in microangiopathies such as diabetic nephropathy and arteriosclerosis [37]. Superoxide dismutase (SOD), which inactivates superoxide using Cu and Zn as cofactors, plays an important role as a protective agent against superoxide [38]; however, its activity in microvascular walls is decreased by glycation due to hyperglycemia [39] and by zinc deficiency [21]. As a result, production of superoxide in the vascular wall tends to increase, resulting in increased formation of peroxynitrite, which in turn leads to a lack of action of the vasodilator NO. This mechanism may be associated with renal microangiopathy in diabetes in the context of zinc deficiency [40]. In addition, as DKD progresses and urinary albumin increases, serum albumin concentration tends to decrease, which decreases the percentage of zinc bound to albumin in the blood and increases that excreted into the urine [12, 13]. In fact, there are reports that urinary zinc excretion in diabetic patients is approximately twice that in healthy controls [41]; urinary zinc excretion is also significantly higher in type 1 diabetes with microalbuminuria than with normal albumin [42].

Previous reports have indicated that low serum zinc levels tend to worsen glycemic control indices [7, 8]. Two proinsulin particles join to form an insulin hexamer and two molecules of zinc are required for this junction [7]. It is assumed that zinc is not only required for the synthesis and secretion of insulin but also involved in the function of the insulin receptor [6, 11]. Zinc deficiency has additionally been suggested to increase insulin resistance [43]. In this study of diabetic patients, serum zinc level was inversely correlated with skeletal muscle index (Table 2), and skeletal muscle index tended to decrease with age (Supplemental Fig. 1). In addition, both males and females with zinc deficiency had skeletal muscle indexes below the diagnostic criteria for sarcopenia (Supplemental Fig. 2). Hypozincemia has been shown significantly associated with frailty in the elderly [44], and muscle mass with serum zinc concentration in patients with chronic liver disease [45]. Zinc deficiency is also associated with frailty and sarcopenia in diabetic patients and may contribute to poor glycemic control. In Japan, 30% of diabetic patients aged 75 years or older are reported to have sarcopenia [46]. In South Korea, where the pathology is considered similar to that in Japan, type 2 diabetes patients have been shown to have decreased limb muscle mass and SMI compared to controls [47]. Zinc is involved in the production of testosterone, which declines with age, predisposing to sarcopenia [48]. In the elderly, high-quality protein intake, which supplies zinc, is important for maintaining skeletal muscle [49]. Elderly diabetic patients are likely to develop decreased insulin sensitivity due to sarcopenia, and it is necessary to pay attention to the intake of zinc-containing foods when providing nutritional guidance to diabetic patients with nephropathy.

### Study limitations

There are some limitations of this research. First, this was a retrospective observational study with a limited number of patients. Prospective studies remain needed to determine whether zinc intake can improve renal function in diabetic patients. Second, the subjects in this study had relatively poor glucose control and most had type 2 diabetes. Further discussion is needed on the universal renal protection effect of zinc intake in diabetic patients with a wide range of pathologies, such as type 1 diabetes. Third, we showed that diabetic nephropathy patients with macroalbuminuria had low serum zinc levels (Fig. 2b), and we speculated that increased urinary Zn excretion was one of the underlying mechanisms. However, urinary zinc excretion could not be measured directly in this retrospective study. Examination of urinary Zn levels may help clarify the cause of decreased serum zinc in the progression of DKD, and further investigation is required on this point.

## Conclusions

This study suggests that zinc deficiency should be considered, especially in patients with DKD beyond overt albuminuria. Furthermore, diabetics with low serum zinc levels exhibited decreased skeletal muscle index. In elderly diabetic patients with DKD, it is desirable to measure the serum zinc concentration so as to prevent adverse events due to zinc deficiency, and to consider zinc supplements and preparations in zinc-deficient patients.

## Supplementary Information

**Additional file 1: Supplemental Fig. 1.** Relationship between skeletal muscle index (SMI) and age in diabetes patients. SMI was determined by the bioelectrical impedance method using a body composition analyzer, adding together the non-fat mass of the upper extremities and that of the lower extremities and dividing by the square of the height. According to the Asian Woking Group for Sarcopenia (AWGS) 2019 diagnostic criteria, an SMI of less than 7.0 kg/m^2^ in men and less than 5.7 kg/m^2^ in women is considered to be at risk for sarcopenia [35]. SMI: skeletal muscle index. Univariate correlation was analyzed using Spearman’s rank correlation. **Supplemental Fig. 2.** Presence or absence of zinc deficiency and SMI levels in diabetic patients. Serum zinc levels were categorized based on the 2018 Clinical Practice Guideline for Zinc Deficiency of the Japanese Society of Clinical Nutrition, and SMI values calculated from body composition were compared. A serum zinc concentration of 80 μg/dL or more was defined as normal, at least 60 μg/dL and less than 80 μg/dL as subclinical zinc deficiency, and less than 60 μg/dL as zinc deficiency. In males, the zinc-deficient group tended to have lower serum zinc concentrations (*P* = 0.012 for trend). In zinc-deficient males, SMI was less than 7.0 kg/m^2^, indicating risk of sarcopenia. SMI: skeletal muscle index. Significance was determined by analysis of covariance. Columns and error bars indicate mean ± standard deviation (S.D).

## Abbreviations

DKD: diabetic kidney disease
CKD: chronic kidney disease
eGFR: estimated glomerular filtration rate
UACR: Urinry albumin-to-creatinine ratio
BMI: Body mass index
SMI: Skeletal muscle mass index
TC: Total cholesterol
TG: Triglyceride
HDL-C: High-density lipoprotein cholesterol
LDL-C: Low-density lipoporotein cholesterol
HbA1c: Hemoglobin A1c
