# Supplementary figures and images for: Association of Serum Zinc Level with severity of chronic kidney disease in diabetic patients: a cross-sectional study
Source: BMC Nephrol. 2022 Dec 21;23:407. doi: 10.1186/s12882-022-03040-x (PMC9769017; doi:10.1186/s12882-022-03040-x)

Supplemental Figure 1

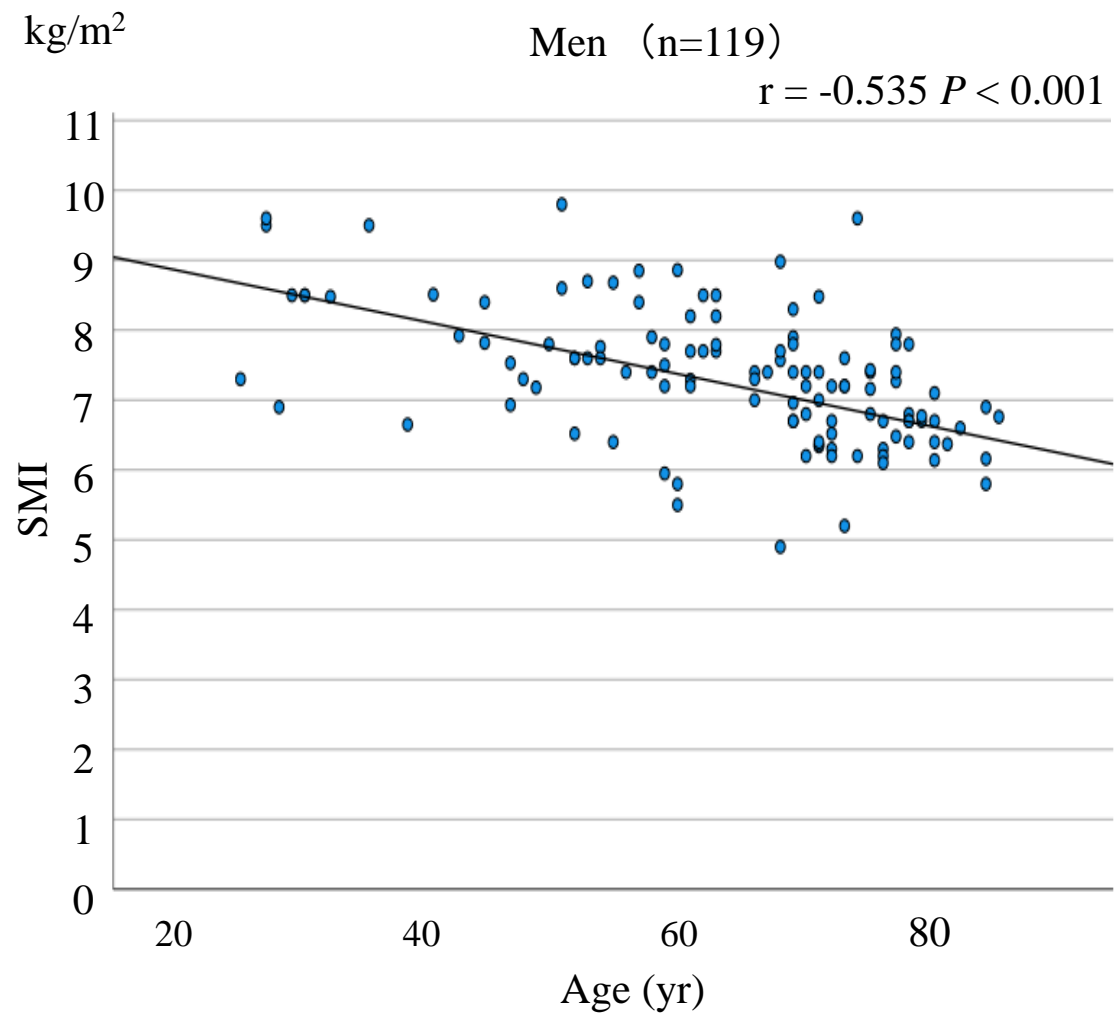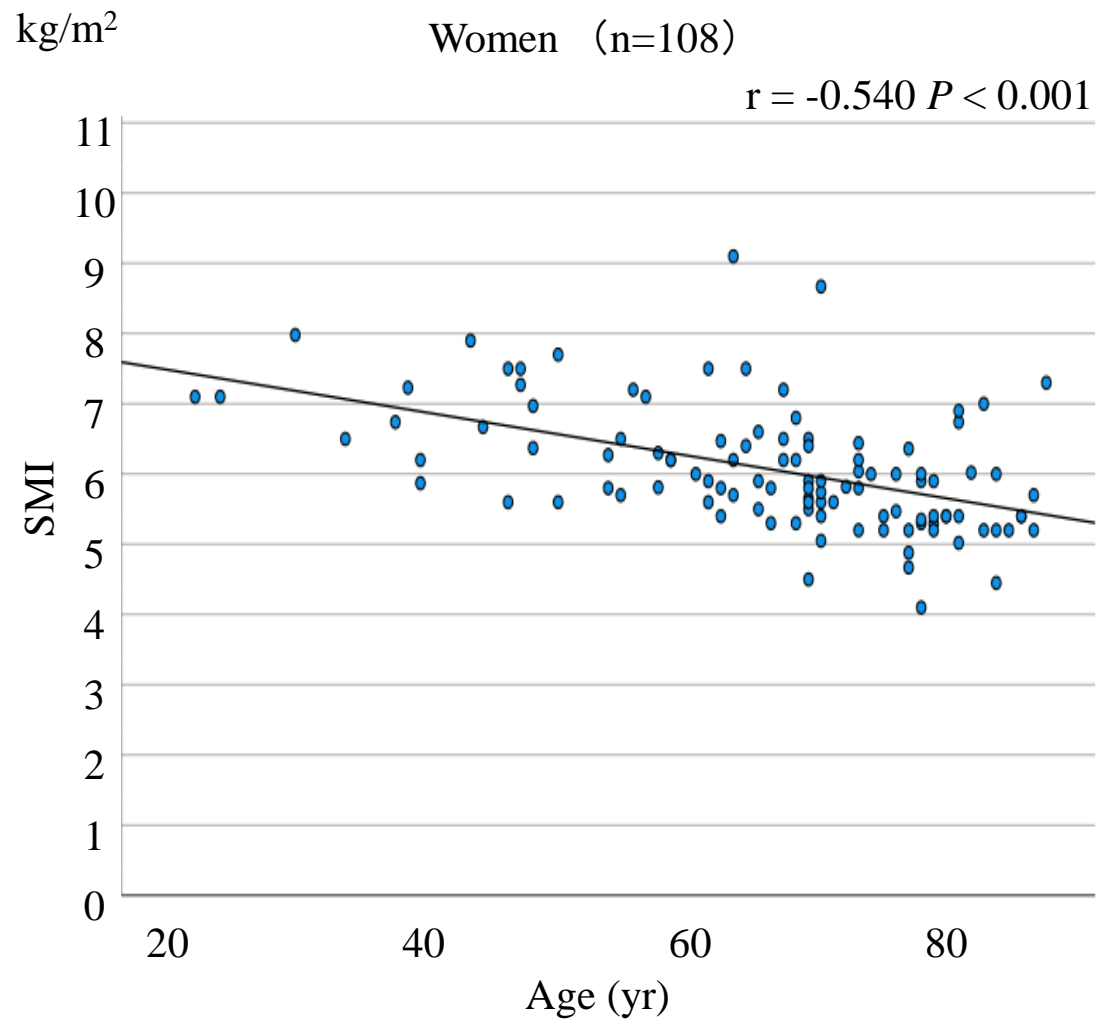

Supplemental Figure 2

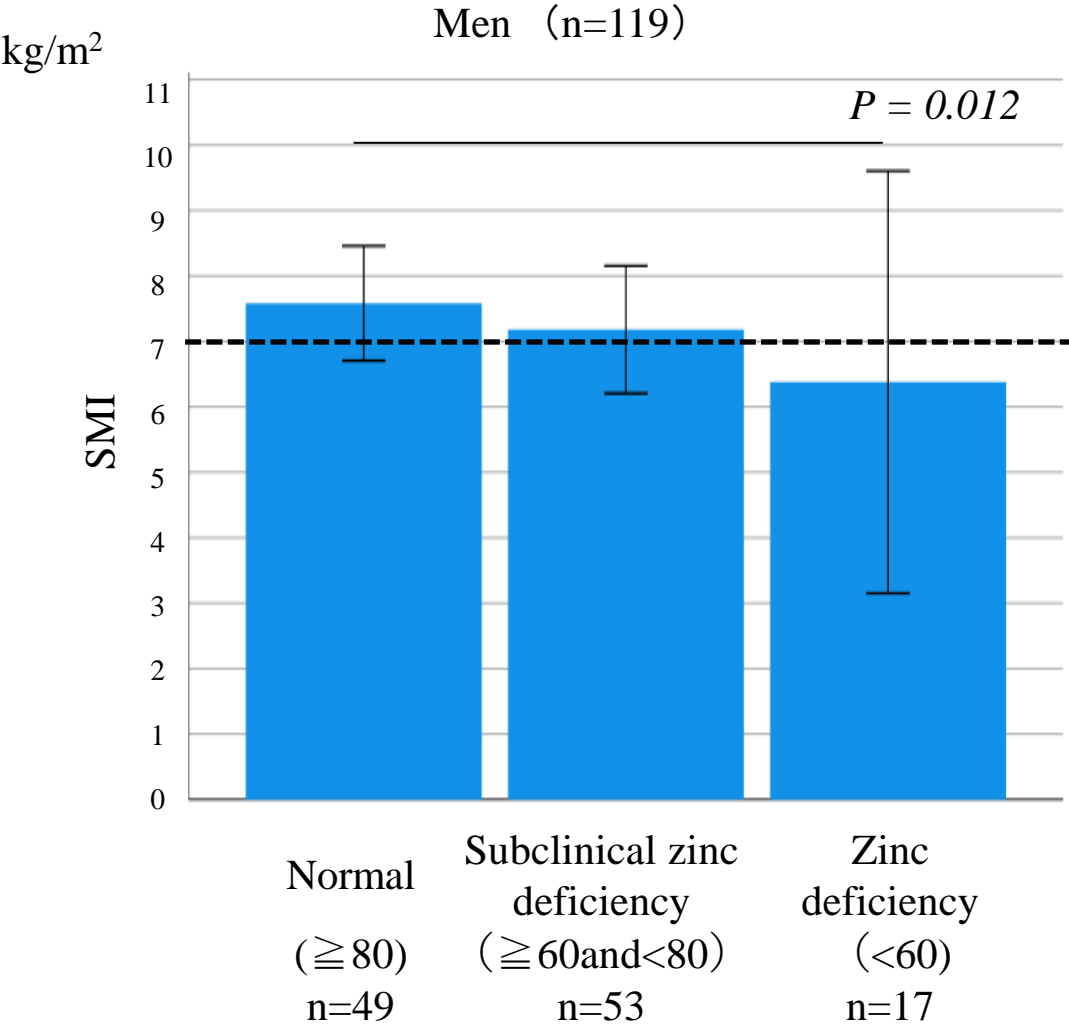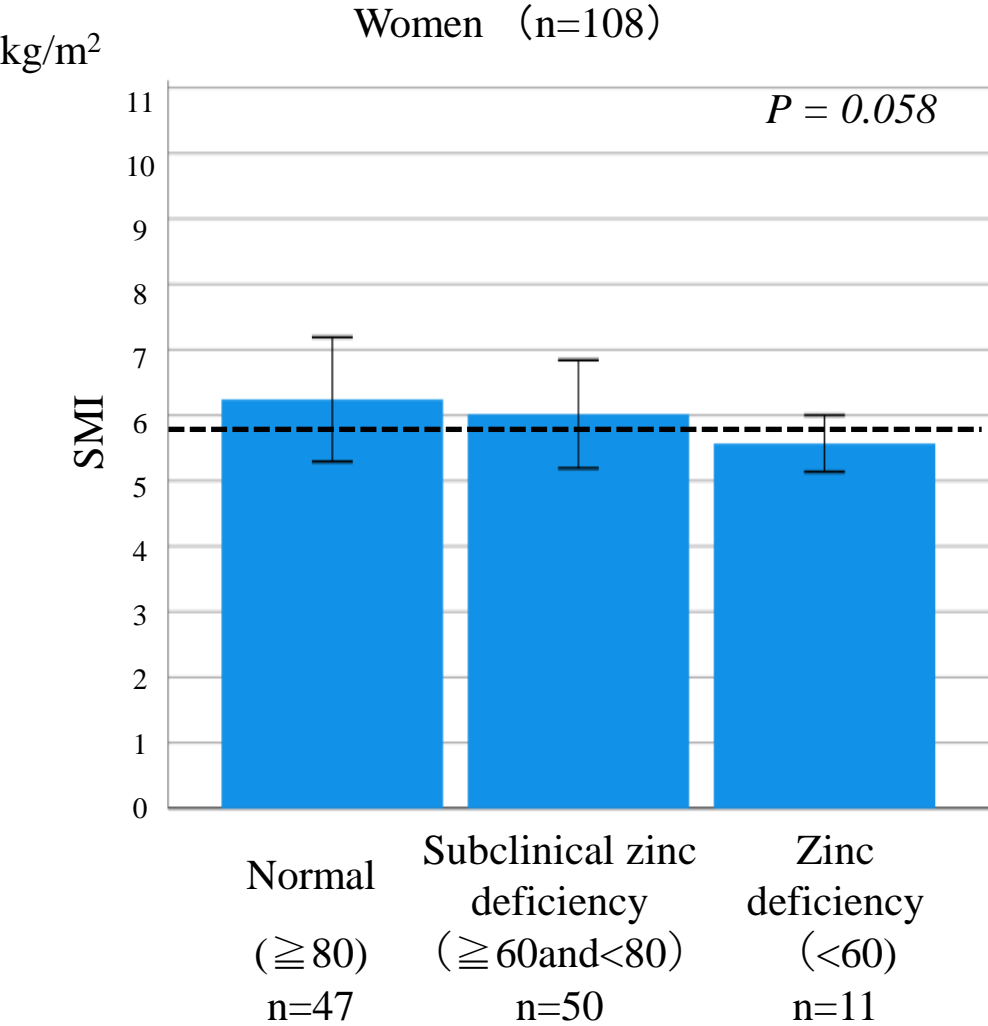

Supplement: Supplementary file 1 — Additional file 1: Supplemental Fig. 1. Relationship between skeletal muscle index (SMI) and age in diabetes patients. SMI was determined by the bioelectrical impedance method using a body composition analyzer, adding together the non-fat mass of the upper extremities and that of the lower extremities and dividing by the square of the height. According to the Asian Woking Group for Sarcopenia (AWGS) 2019 diagnostic criteria, an SMI of less than 7.0 kg/m2 in men and less than 5.7 kg/m2 in women is considered to be at risk for sarcopenia [35]. SMI: skeletal muscle index. Univariate correlation was analyzed using Spearman’s rank correlation. Supplemental Fig. 2. Presence or absence of zinc deficiency and SMI levels in diabetic patients. Serum zinc levels were categorized based on the 2018 Clinical Practice Guideline for Zinc Deficiency of the Japanese Society of Clinical Nutrition, and SMI values calculated from body composition were compared. A serum zinc concentration of 80 μg/dL or more was defined as normal, at least 60 μg/dL and less than 80 μg/dL as subclinical zinc deficiency, and less than 60 μg/dL as zinc deficiency. In males, the zinc-deficient group tended to have lower serum zinc concentrations (P = 0.012 for trend). In zinc-deficient males, SMI was less than 7.0 kg/m2, indicating risk of sarcopenia. SMI: skeletal muscle index. Significance was determined by analysis of covariance. Columns and error bars indicate mean ± standard deviation (S.D). [file 12882_2022_3040_MOESM1_ESM.pdf]
